# VopX, a novel *Vibrio cholerae* T3SS effector, modulates host actin dynamics

**DOI:** 10.1128/mbio.03018-24

**Published:** 2025-01-29

**Authors:** Megan Ulbrich, Christopher H. Seward, Andrei I. Ivanov, Brian M. Ward, J. Scott Butler, Michelle Dziejman

**Affiliations:** 1 Department of Microbiology and Immunology, University of Rochester Medical Center, Rochester, New York, USA; 2 Department of Inflammation and Immunity, Lerner Research Institute, Cleveland Clinic Foundation, Cleveland, Ohio, USA; Emory University School of Medicine, Atlanta, Georgia, USA

**Keywords:** *V. cholerae*, T3SS, effector, cytoskeleton, pathogenesis

## Abstract

Pathogenic *Vibrio cholerae* strains cause cholera using different mechanisms. O1 and O139 serogroup strains use the toxin-co-regulated pilus (TCP) and cholera toxin (CT) for intestinal colonization and to promote secretory diarrhea, while non-O1/non-O139 serogroup strains are typically non-toxigenic and use alternate virulence factors to cause a clinically similar disease. An O39 serogroup, TCP/CT-negative *V. cholerae* strain, named AM-19226, uses a type III secretion system (T3SS) to translocate more than 10 effector proteins into the host cell cytosol. Effectors VopF and VopM directly interact with the host actin and contribute to colonization. Our previous studies using the *Saccharomyces cerevisiae* model system identified VopX as a third effector that alters cytoskeletal dynamics. Herein, we used complementary approaches to translate yeast findings to a mammalian system and determined the target and mechanism of VopX activity. VopX overexpression in HeLa cells caused dramatic cell rounding. Co-culture of strain AM-19226 with polarized Caco-2/BBE monolayers increased formation of stress fibers and focal adhesions, as well as Caco-2/BBE adherence to extracellular matrix in a VopX-dependent manner. Finally, we demonstrate *in vitro* that VopX can act as a guanine nucleotide exchange factor for RhoA, which functions upstream of a mitogen-activated protein kinase (MAPK) signaling pathway regulating cytoskeletal dynamics. Our results suggest that VopX activity initiates a signaling cascade resulting in enhanced cell-extracellular matrix adhesion, potentially preventing detachment of host cells, and facilitating sustained bacterial colonization during infection. VopX function is therefore part of a unique pathogenic strategy employed by T3SS-positive *V. cholerae*, which involves multiple cytoskeletal remodeling mechanisms to support a productive infection.

**IMPORTANCE:**

Despite different infection strategies, enteric pathogens commonly employ a T3SS to colonize the human host and cause disease. Effector proteins are unique to each T3SS-encoding bacterial species and generally lack conserved amino acid sequences. However, T3SS effectors from diverse pathogens target and manipulate common host cell structures and signaling proteins, such as the actin cytoskeleton and MAPK pathway components. T3SS-encoding *Vibrio cholerae* strains and effectors have been relatively recently identified, and the mechanisms used to mediate colonization and secretory diarrhea are poorly understood. Two *V. cholerae* effectors that modify the host actin cytoskeleton were shown to be important for colonization. We therefore sought to determine the target(s) and mechanism of a third actin-reorganizing effector, VopX, based on results obtained from a yeast model system. We recapitulated actin-based phenotypes in multiple mammalian model systems, leading us to identify the molecular function of the *V. cholerae* VopX effector protein.

## INTRODUCTION

Gram-negative bacterial pathogens commonly employ type III secretion systems (T3SSs) as essential virulence factors, which have been studied for decades (1–5). Despite sharing a conserved structural secretion apparatus and translocation mechanism, the arsenal of effector proteins transported directly into the host cell is typically unique to each species (6–8). Effector proteins are diverse at both the amino acid and functional levels and interact with a wide array of host cell proteins, pathways, and processes (9–12). Effectors thus mediate distinct, species-specific host colonization and virulence strategies. Consequently, identifying the functions of individual bacterial effector proteins is essential for understanding the molecular mechanisms responsible for pathogenesis.

*Vibrio cholerae* is a long-studied enteric pathogen that causes the secretory diarrheal disease cholera. However, *V. cholerae* T3SS-mediated pathogenesis was discovered relatively recently (13–15). The toxin co-regulated pilus (TCP) and cholera toxin (CT) are canonical and well-characterized virulence factors essential for colonization and secretory diarrhea, but in a subset of TCP/CT-negative strains, a T3SS is responsible for causing a clinically similar disease (16–21). Different *V. cholerae* virulence mechanisms can be categorized and partially segregated by serogroup designation and common epidemiological properties. Epidemic-causing O1 and O139 serogroup strains colonize and cause disease exclusively using TCP and CT-mediated mechanisms. Conversely, *V. cholerae* non-O1/non-O139 serogroup strains, responsible for global and sporadically occurring infections, use diverse and pilus/toxin-independent pathogenic mechanisms, including the T3SS and translocated effector molecules (14, 22).

Non-O1/non-O139 serogroup *V. cholerae* strains are an understudied group of human pathogens responsible for clinically significant disease that includes both enteric and extraintestinal infections (23–25). Estimates suggest that approximately 30%–40% of non-O1/non-O139 serogroup strains encode a T3SS (22). Thus, the ability to cause a clinically similar disease using fundamentally different molecular mechanisms emphasizes the need to understand *V. cholerae* T3SS effector protein activities. Furthermore, effectors are likely responsible for mediating unknown host-pathogen molecular interactions that can inform our understanding of how human intestinal tissues maintain homeostasis.

Despite the lack of sequence similarity, T3SS effector protein activities often converge on conserved cellular processes and pathways and function as “molecular mimics” when interacting with host cell proteins (26–28). For example, the actin cytoskeleton is an important T3SS effector target (29–32). Effectors have been shown to interact directly with actin monomers or actin filaments (33, 34). Signaling pathways controlling actin dynamics also serve as common effector targets to promote activities essential for pathogenesis, such as colonization. For example, *Salmonella* spp., pathogenic *Escherichia coli* (enteropathogenic and enterohemorrhagic *E. coli*), and *Yersinia* spp. each deliver effectors to the host cell that modulate actin dynamics and perform functions required for establishing a colonization niche (29, 35–37). Overlapping effector targets from such diverse species therefore highlight the importance of actin manipulation in T3SS-mediated infection strategies.

AM-19226 is a clinically isolated, O39 serogroup, TCP/CT-negative, T3SS-positive *V. cholerae* strain (19, 20, 38). The T3SS is essential for colonization and secretory diarrhea, and AM-19226 has served as a prototypical strain for understanding T3SS effector protein function and mechanisms leading to TCP/CT-independent disease. Of the 13 secreted AM-19226 effectors (Vops), two Vops were identified thus far as targeting the host cytoskeleton: VopF, which contains WASP/formin homology domains and nucleates actin, and VopM, which bundles actin filaments (20, 39–41). VopF and VopM are essential for colonization in infant mouse and infant rabbit models of infection (42, 43). However, the downstream consequences of actin rearrangements, additional effectors modulating actin filament dynamics, and the contribution of actin reorganization to AM-19226 pathogenic mechanisms remain to be elucidated.

We previously identified VopX as a novel, 252-amino acid effector protein with a secondary structure prediction composed of six or seven alpha helices (38). *In vitro*, VopX was translocated into eukaryotic cells with an efficiency comparable to VopM (38). Initial experiments to identify putative targets of VopX interactions used *Saccharomyces cerevisiae* as a genetically tractable model system, and results suggest that components of the mitogen-activated protein kinase (MAPK) cell wall integrity (CWI) signaling pathway were the target(s) of VopX activity (44). The yeast CWI pathway is a signaling hierarchy that affects transcriptional responses to cell wall stress and includes the terminal transcriptional regulator Rlm1 with upstream MAPK proteins, whose activity is governed by Rho1 (45–47). Combined with results indicating actin perturbations and halted cell cycle progression, we hypothesized that VopX functions during infection to modulate signaling events targeting actin cytoskeletal structure and dynamics (44).

Our previous work, however, did not investigate VopX activity in mammalian cells. In the present study, we turned to mammalian cell model systems to determine whether VopX modulates the actin cytoskeleton. Our results led us to identify VopX-dependent phenotypes that likely contribute to a productive infection. *In vitro* studies with purified protein demonstrate that VopX can activate the RhoA small GTPase, providing a mechanistic explanation for VopX-induced phenotypes and identifying VopX as a third *V. cholerae* effector protein capable of modulating actin filament dynamics using molecular mimicry.

## RESULTS

### VopX overexpression produces morphological and cytoskeletal changes in mammalian cells

Since we previously observed VopX-induced growth inhibition and altered actin filament dynamics during budding in yeast (44), we examined morphological and cytoskeletal phenotypes after overexpressing VopX in mammalian cells using a *Vaccinia* virus/T7 RNA polymerase hybrid system (48). We constructed a plasmid to express the VopX coding sequences fused to a C-terminal HA tag under bacteriophage T7 RNA polymerase promoter control (pVopX-HA). pVopX-HA was transfected into HeLa cells infected with *Vaccinia* virus vTF7-3 in the presence of cytosine arabonoside (49). The addition of cytosine arabonoside restricts virus replication and allows only early viral gene expression. The combined plasmid and virus features thus ensure that *vopX-HA* is transcribed and translated with high efficiency, typically in >75% of cells. Transfection was performed at a relatively low efficiency (<50%), allowing comparison of cells both expressing and not expressing VopX-HA in the same field of view.

**Fig 1:**
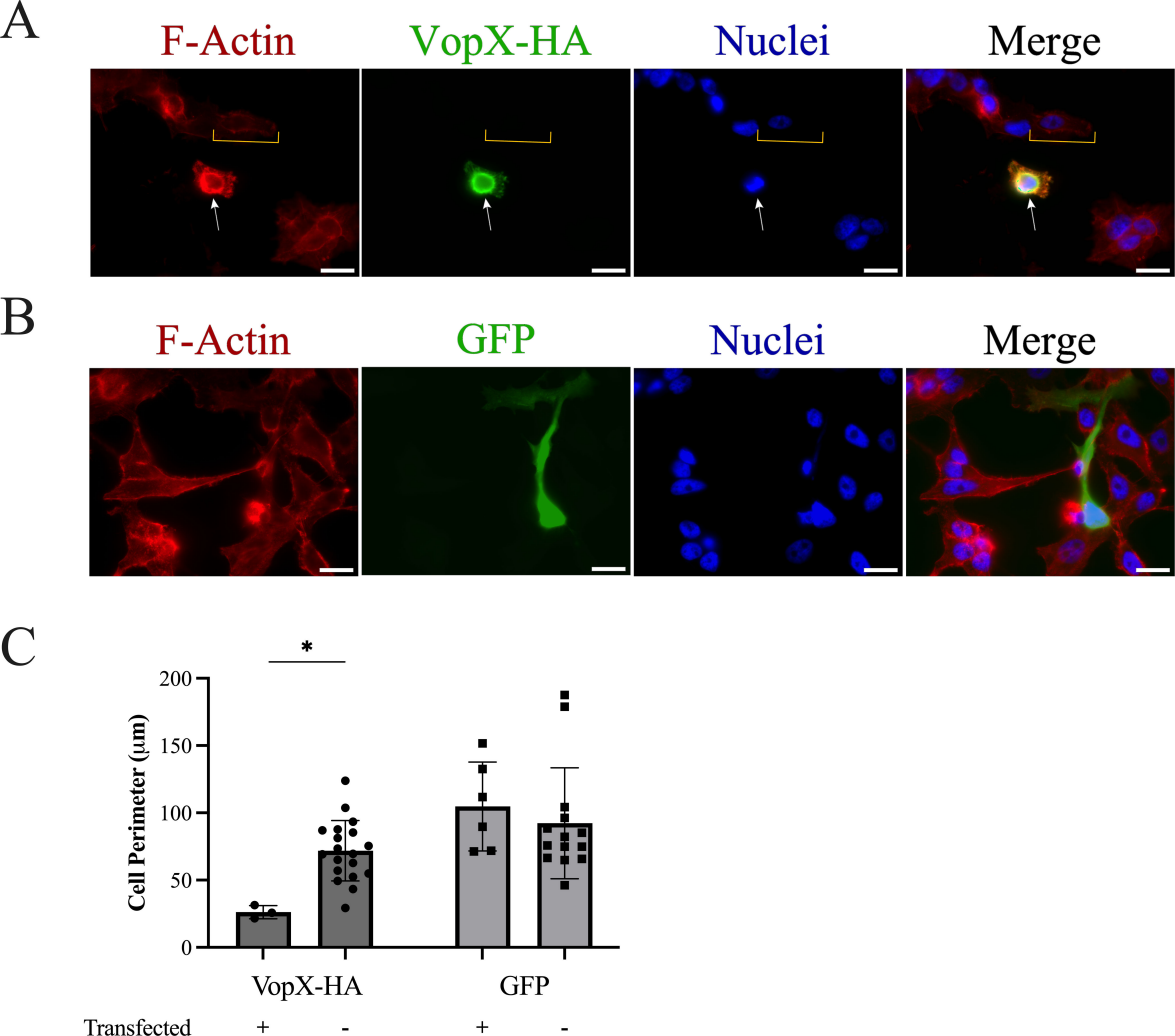
VopX overexpression in HeLa cells results in cell shape changes. (A) Immunofluorescence imaging of HeLa cells infected with *Vaccinia* virus vTF7-3/RFP-A4at a multiplicity of infection of 0.5 and transiently transfected with a vector expressing VopX-HA. Cells were fluorescently labeled for F-actin with Alexa Fluor 647 phalloidin (red), nuclei with 4′,6-diamidino-2-phenylindole (blue), and HA detected using an anti-HA antibody (green). White arrows indicate a transfected cell and yellow brackets indicate an untransfected cell. (B) Parallel experiment of HeLa cells infected with vTF7-3/RFP-A4 and transiently transfected with pGFP. Scale bars represent 20 μm. (C) Quantification of cell perimeter from transfected and untransfected HeLa cells. Measurements are pooled from three images taken from a single biological replicate. Statistics were generated using two-way analysis of variance with Šídák’s multiple comparison test. *P ≤ 0.05. VopX-HA, transfected *n* = 3; VopX-HA, transfected *n* = 19; GFP, transfected *n* = 6; GFP, untransfected *n* = 14. The study was conducted three times and produced similar results.

Figure 1A shows fluorescence labeling of vTF7-3 infected HeLa cells transfected with pVopX-HA. VopX-expressing cells identified by the HA tag were characterized by an atypical, rounded morphology, based on the F-actin labeling (white arrows). In contrast, neighboring cells that do not express VopX showed F-actin staining consistent with the elongated shape characteristic of HeLa cells (Fig. 1A, yellow brackets). Strikingly, we observed VopX localization using the HA tag at the actin-rich periphery associated with an irregular cell membrane. We repeated the assay using a vector expressing GFP (pGFP) to determine whether overexpression of an unrelated protein could be responsible for the morphological changes. As shown in Fig. 1B, the GFP signal is distributed evenly throughout the cell, and GFP overexpression did not produce cell rounding or actin-rich cell surface protrusions. To determine if viral infection altered cell morphology, we performed a parallel experiment with a vTF7-3 virus that had the *Vaccinia* A4 core protein translationally fused to mKate2 (vTF7-3/RFP-A4) (Fig. S1). Cells infected with vTF7-3/RFP-A4 alone (and not transfected by pVopX-HA) retained the elongated HeLa morphology and did not develop irregular cell membrane morphologies or protrusions, whereas those transfected and expressing VopX-HA were rounded, consistent with results shown in Fig. 1A.

Using the phalloidin florescent signal to identify cell borders delineated by cortical actin filaments, we measured the perimeter of pVopX-HA and pGFP transfected cells and control cells (not transfected) to provide a quantitative measure of cell rounding. The results of individual cell measurements are shown in Fig. 1C. As described earlier, performing the low-efficiency transfection allowed perimeter measurements to be taken for cells both expressing and not expressing VopX-HA or GFP, although it resulted in fewer transfected cells available within any given image. Nonetheless, cells expressing VopX-HA had a three- to fourfold lower perimeter measurement compared to cells not transfected with the pVopX-HA plasmid and cells transfected with the plasmid.

### T3SS-delivered VopX alters cell morphology in polarized epithelial monolayers

**Fig 2:**
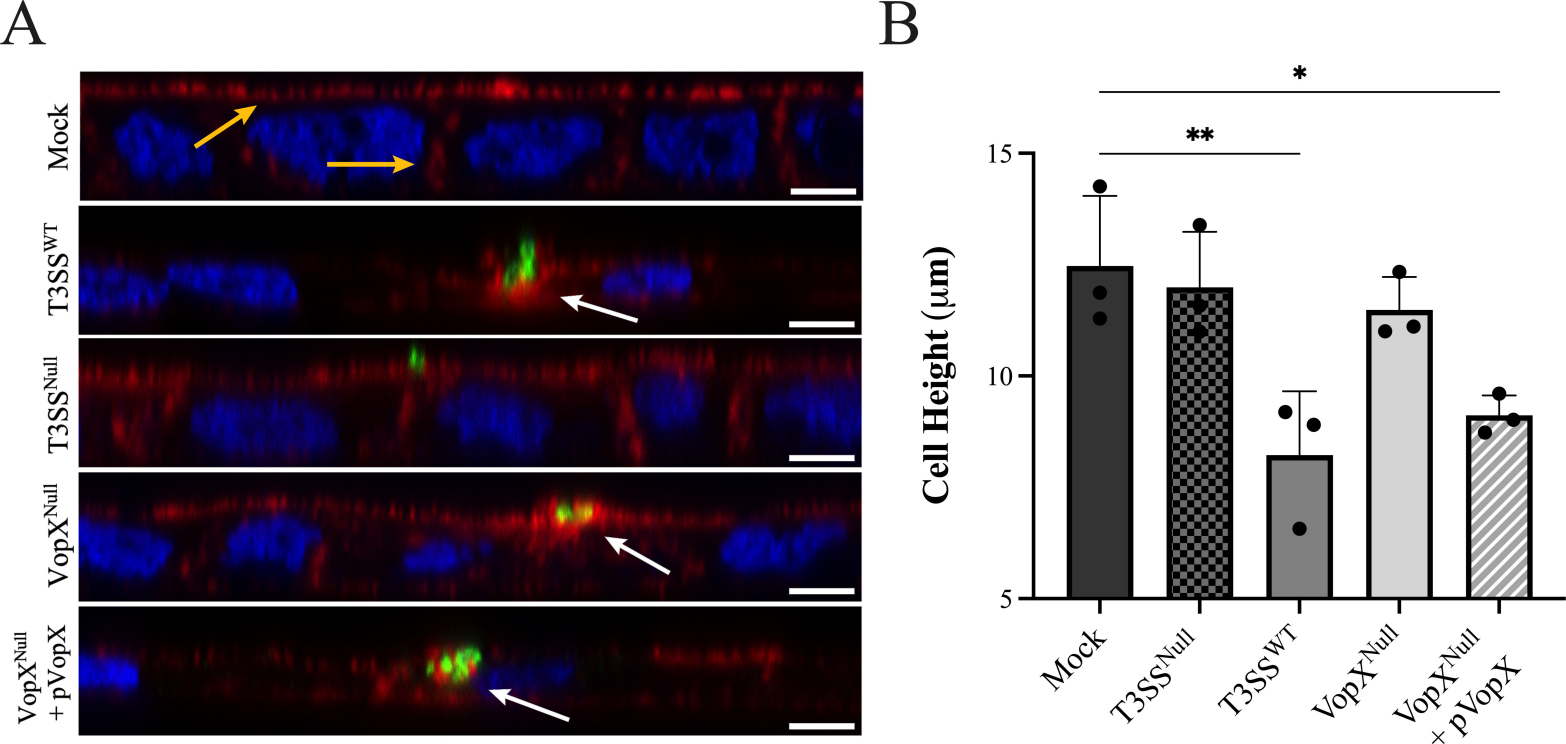
VopX activity reduced Caco-2/BBE cell height during AM-19226 co-culture. (A) Confocal Z stacks viewed from the XZ plane showing differentiated Caco-2/BBE co-cultured with the indicated AM-19226 strain at a multiplicity of infection of ~10 or phosphate-buffered saline (PBS; mock) for 3 h. Cells were fluorescently labeled for F-actin with Alexa Fluor 647 phalloidin (red), nuclei (4′,6-diamidino-2-phenylindole, blue), and *V. cholerae* detected using a polyclonal rabbit antibody developed against whole AM-19226 cells (green). Yellow arrows indicate apical and lateral F-actin signal. White arrows indicate actin-rich signal below bacteria. Scale bars represent 5 μm. (B) Quantification of cell height in microns. Strains are noted on the *X* axis. Cell height was determined by measuring the length of the actin signal in the *Z* plane. Data represent the average ± standard deviation of three biological replicates. Statistics were generated by ordinary one-way analysis of variance with Dunnett’s multiple comparison test. *P ≤ 0.05, **P ≤ 0.01. The study was conducted three times and produced similar results.

Given that actin reorganization and cell rounding were clear phenotypes of HeLa cells expressing VopX, we next sought to investigate the effects of VopX on cell morphology and the actin cytoskeleton in an intestinal cell tissue culture model. We grew polarized monolayers of brush-border forming Caco-2/BBE human intestinal epithelial cells on Transwell membrane filters to mimic small intestinal epithelium. We co-cultured the mature cell monolayers with isogenic AM-19226 strains so that effectors would be expressed and delivered under “native” conditions. Differentiated Caco-2/BBE monolayers were co-cultured with T3SS-*wt* or T3SS-*null* AM-19226 strains for 3 h and subjected to dual fluorescence labeling for F-actin and attached bacteria. An *xz* projection of confocal images shows a typical columnar morphology of mock co-cultured Caco-2/BBE monolayers with prominent cortical F-actin labeling at the cell apex and lateral cell-cell contact areas (Fig. 2A; mock panel, yellow arrows). In contrast, Caco-2/BBE monolayers co-cultured with an AM-19226 strain encoding a functional T3SS (T3SS^WT^) display marked morphological abnormalities exhibited by a visibly decreased cell height (Fig. 2A). Quantitative analysis of confocal images indicated that the height of AM-19226 infected cell monolayers was reduced by ~50% from 11 - 14 μm to 6–10 μm (Fig. 2B). Interestingly, the decreased height was not restricted to epithelial cells directly interacting with bacteria. The alteration was also evident in adjacent cells within the monolayer where we did not observe adherent bacteria, suggesting an integrated response of the entire Caco-2/BBE monolayer that most likely involves mechanosensing/transduction events.

The morphological abnormalities of *Vibrio*-associated Caco-2/BBE cells were accompanied by the collapse of lateral F-actin structures and accumulation of actin filaments in close proximity to attached bacteria (Fig. 2A, white arrows). The actin-rich signal observed under bacteria is consistent with reports describing an actin bundling activity attributed to the VopM effector protein (40). Notably, the monolayer co-culture with the AM-19226 T3SS^Null^ strain, which did not express a functional secretion system and was unable to translocate effector proteins, appeared similar to the mock-infected monolayers and preserved epithelial cell height and lateral actin filament structures (Fig. 2A and B). We therefore concluded that a functional T3SS, and hence effector protein activity, was required for the dramatic morphological changes and cytoskeletal abnormalities.

To determine whether VopX was required for the T3SS-mediated phenotype, we co-cultured Caco-2/BBE cells with an AM-19226 VopX^Null^ isogenic strain. The isogenic strain expresses a functional secretion system with a nonsense mutation in the VopX coding sequence (41). Cells did not exhibit the AM-19226 T3SS^WT^ co-culture phenotype and instead showed a preserved epithelial cell height and lateral F-actin labeling characteristic of that observed for mock co-cultured monolayers and those co-cultured with the AM-19226 T3SS^Null^ strain (Fig. 2A and B). Note that the VopM-dependent F-actin-rich staining under the bacteria remained detectable in the VopX^Null^ panel. Co-culture using an AM-19226 VopX^Null^ strain, where VopX was provided in *trans* expressed from an arabinose-inducible promoter (VopX^Null^ + pVopX), demonstrated that the phenotype was due to the absence of VopX protein, since we observed a decreased intensity of cortical and lateral actin staining and a reduction in cell height, similar to that observed during co-culture with the AM-19226 T3SS^WT^ strain (Fig. 2A and B). We therefore concluded that, consistent with the VopX-dependent phenotypes using the viral infection/transfection system, delivery of VopX by the T3SS resulted in altered epithelial cell morphology and actin cytoskeletal remodeling. VopX therefore appears to be necessary for the dramatic phenotypes observed during AM-19226 infection of a polarized intestinal cell monolayer.

### VopX activity reorganizes different actin structures in polarized epithelial cells

**Fig 3:**
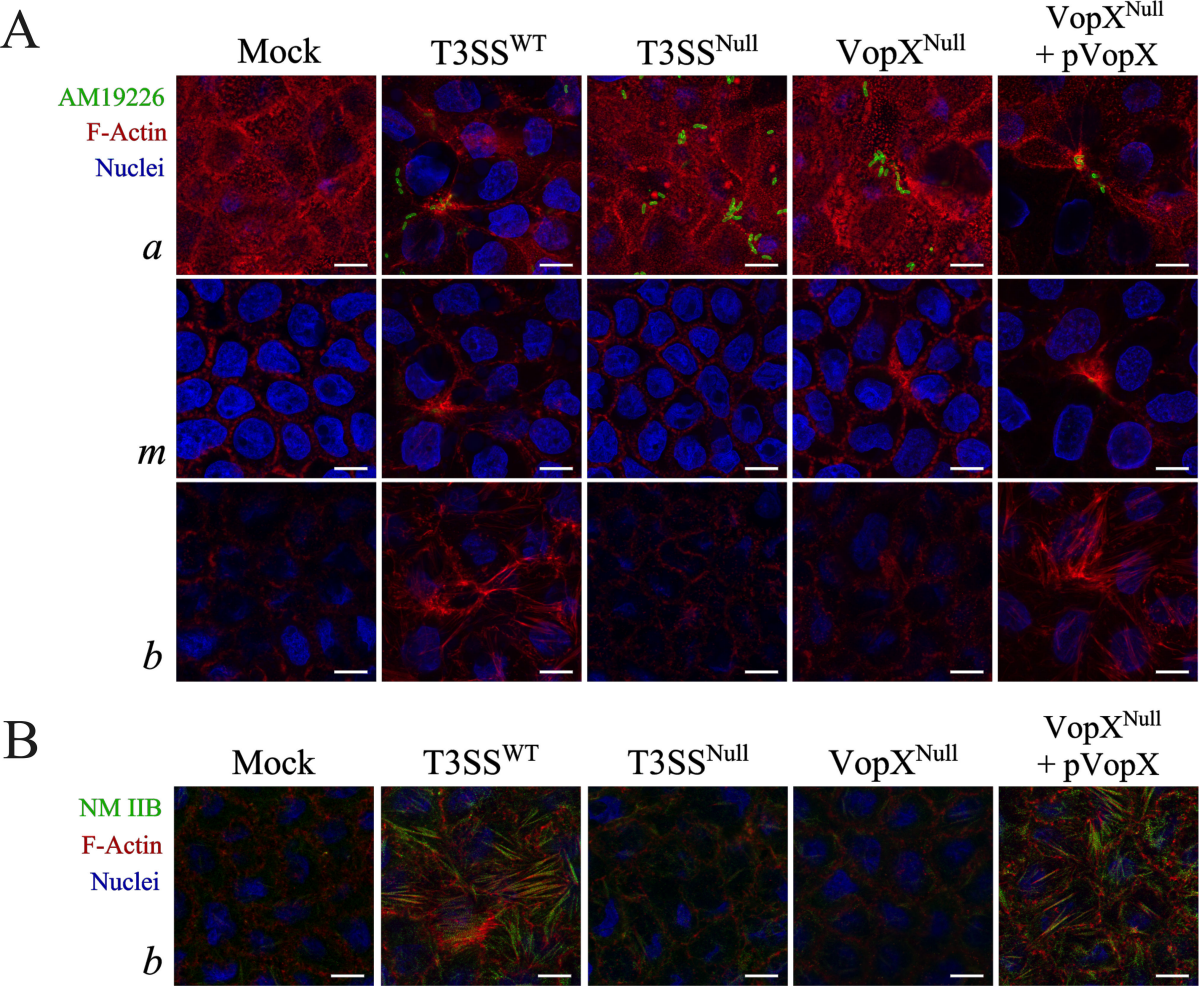
VopX is required for stress fiber formation in polarized Caco-2/BBE cells. (A) Three planes, taken at 0.30 μm step size, shown as maximum intensity projections of differentiated Caco-2/BBE monolayers co-cultured with the indicated AM-19226 strain for 3 h (multiplicity of infection of ~10 or phosphate-buffered saline). The top row shows the apical cell surface (*a*); the middle row shows them midpoint of the cells (*m*); and the bottom row shows the basal cell surface (*b*). F-actin was labeled with Alexa Fluor 647 phalloidin (red); nuclei were labeled with 4′,6-diamidino-2-phenylindole (DAPI) (blue); and bacteria were detected using a polyclonal rabbit antibody developed against whole cell AM-19226 (green). Scale bars represent 10 μm. (B) Parallel experiment involving co-labeling of F-actin with Alexa Fluor 647 phalloidin (red), nuclei (DAPI, blue), and actin motor, NM IIB (green). Images depict the basal cell surface. Scale bars represent 10 μm. The study was conducted three times and produced similar results.

We further investigated the effect of VopX on cytoskeletal organization during AM-19226 infection of Caco-2/BBE polarized monolayers by performing F-actin imaging at three focal planes: the apical cell surface, the basal cell surface, and the midpoint between the apical and basal cell planes. As shown in Fig. 3A, the apical plane of the mock co-culture was characterized by a dense actin filament network indicative of the cortical F-actin belt and the terminal web with microvilli roots. The middle confocal plane exhibited prominent cortical F-actin structures associated with lateral cell-cell contacts, whereas the basal plane showed diminished lateral F-actin labeling. Infection of Caco-2/BBE cells with the AM-19226 T3SS^WT^ strain resulted in marked alterations of the actin cytoskeleton at distinct cellular locations. Specifically, bacterial infection decreased apical F-actin staining, concurrent with increased assembly of basal actin filaments (Fig. 3A; compare “*a*” panels to “*b*” panels). Notably, the basal F-actin structures appeared as thick parallel actin filament bundles resembling stress fibers. We also observed dense actin staining under the bacteria within the apical (*a*) and midpoint (*m*) images, likely resulting from VopM activity as previously described (40). Importantly, we did not observe increased basal F-actin staining after co-culture with the AM-19226 T3SS^Null^ or AM-19226 VopX^Null^ strains (Fig. 3A). Reduced apical staining and increased basal staining were both restored by expressing VopX in *trans* using the AM-19226 VopX^Null^ + pVopX strain.

To determine if the basal actin structures induced by co-culture with the T3SS^WT^ strain were indeed stress fibers, we performed dual fluorescence labeling experiments where we stained for F-actin and non-muscle myosin IIB (NM IIB) (Fig. 3B). NM IIB is an actin motor important for the formation of stress fibers in multiple cell types (50–53). We observed co-localization of F-actin and NM IIB staining at the basal cell surface, providing strong evidence that the basal actin-rich fibers are stress fibers (Fig. 3B, red and green channels). Consistent with previous results, co-culture with phosphate-buffered saline (PBS; mock), AM-19226 T3SS^Null^, and AM-19226 VopX^Null^ strains exhibited minimal NM IIB staining at the epithelial cell base, whereas co-culture with AM-19226 T3SS^WT^ and AM-19226 VopX^Null^ + pVopX showed accumulation of the actin stress fibers associated with NM IIB (Fig. 3B).

### VopX-dependent stress fiber formation modulates Caco-2/BBE cell adherence

**Fig 4:**
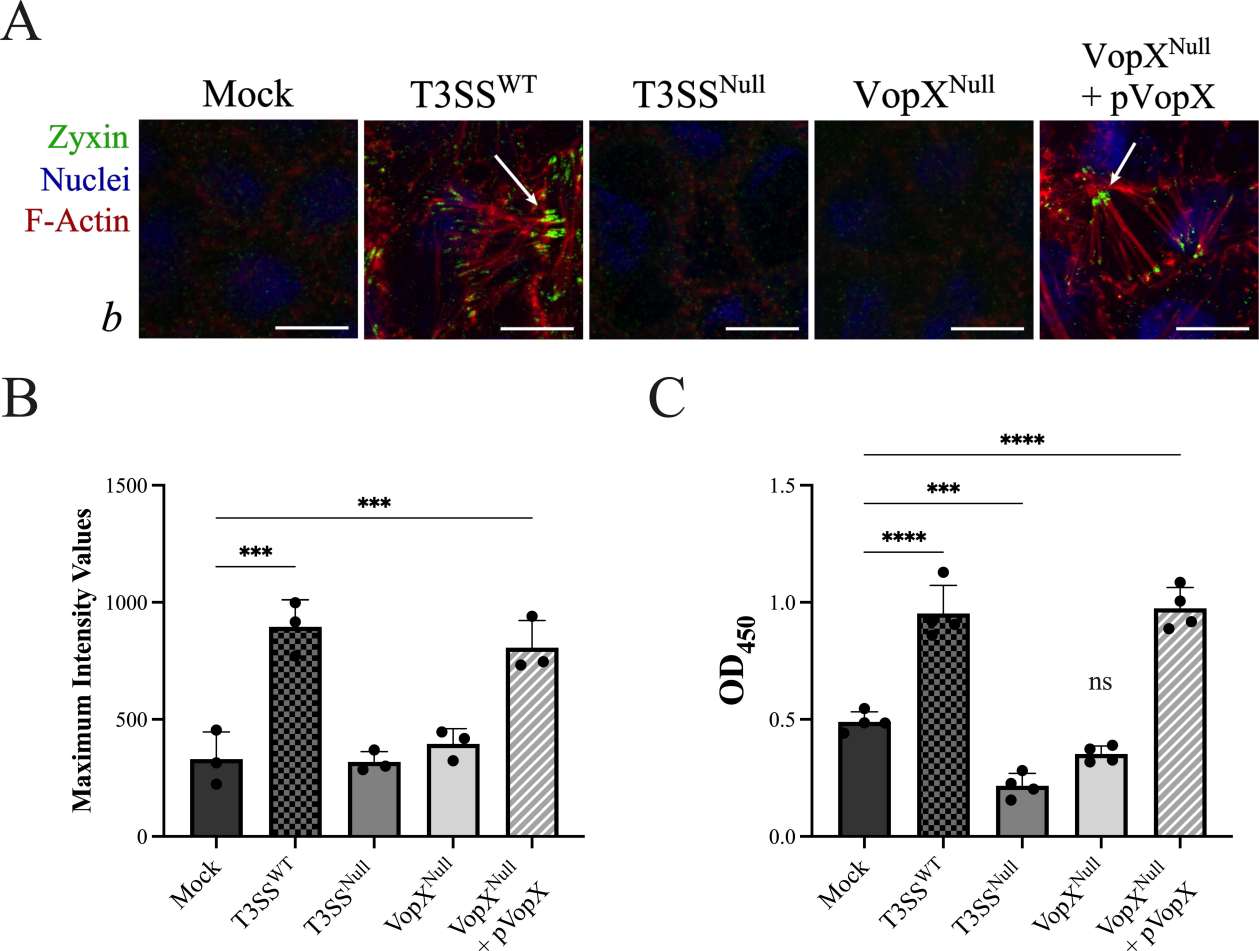
VopX activity increases Caco-2/BBE adherence to the extracellular matrix. (A) Maximum intensity projections of three planes, taken at 0.30 μm step size, showing differentiated Caco-2/BBE monolayers co-cultured with the indicated AM-19226 strain for 3 h (multiplicity of infection ~10 or PBS). Cells were fluorescently labeled for F-actin with Alexa Fluor 647 phalloidin (red), nuclei (DAPI, blue), and zyxin (green). Images depict the basal cell surface. Scale bars represent 10 μm. (B) Maximum intensity values of zyxin staining from three 0.9 μm confocal projections. Data represent the average of three biological replicates. Statistics were generated by ordinary one-way analysis of variance (ANOVA) with Dunnett’s multiple comparison test. ***P ≤ 0.001. (C) Optical density at 450 nm of Caco-2/BBE cells stained with crystal violet, following an inverted spin after 3 h co-culture with the indicated AM-19226 strain or PBS (mock). Data represent the average ±standard deviation of four technical replicates. Statistics were generated by ordinary one-way ANOVA with Dunnett’s multiple comparison test. ***P ≤ 0.001, ****P ≤ 0.0001. The study was conducted three times with similar results. ns, not significant.

Further evidence supporting stress fiber formation was collected by staining using an antibody recognizing zyxin. Zyxin is an essential component of mature focal adhesions (FAs), which mediate cell attachment to the extracellular matrix by participating in multiprotein complexes at the matrix adhesion sites linked to the actin stress fibers (54). In Fig. 4A, we observed punctate zyxin staining at the poles of the F-actin structures when monolayers were co-cultured with AM-19226 T3SS^WT^ and AM-19226 VopX^Null^ + pVopX strains. Conversely, mock, AM-19226 T3SS^Null^, and AM-19226 VopX^Null^ co-cultures all displayed minimal basal cell surface staining.

Zyxin staining was quantified using maximum intensity values of three fields of view from one experiment, and the results are shown in Fig. 4B. Zyxin intensity is greatest in co-culture experiments with the AM-19226 T3SS^WT^ strain and, notably, decreases to a level similar to that observed for mock-infected monolayers when either the AM-19226 T3SS^Null^ or AM-19226 VopX^Null^ strains are used. Again, complementation of the AM-19226 VopX^Null^ strain restored levels of zyxin staining intensity similar to those observed for the AM-19226 T3SS^WT^strain. We thus concluded that VopX is required for FA assembly during infection.

We next hypothesized that increased FA formation would lead to enhanced Caco-2/BBE cell attachment to the extracellular matrix. In Fig. 4C, we present the results of an adherence assay for Caco-2/BBE cells co-cultured with wild-type or mutant AM-19226 strains in a 96-well plate. Under standard plating condition, eukaryotic cells will use vitronectin and fibronectin available in fetal calf serum as the initial attachment matrix (55). Following our standard 3 h co-culture protocol at a multiplicity of infection (MOI) of ~10, we centrifuged the inverted plate and then stained remaining cells with crystal violet using optical density measurement as a reporter of cell adhesion. Compared to the mock co-culture condition, co-culture with the AM-19226 T3SS^WT^ strain resulted in a twofold increase in cell attachment as measured by optical density at 450 nm (OD_450_). Interestingly, co-culture with the AM-19226 T3SS^Null^ strain reduced adherence ~2-fold compared to mock co-culture (*P* ≤ 0.0001). Co-culture with the AM-19226 VopX^Null^ strain also reduced adherence and could be complemented to levels similar to that observed for co-culture with the AM-19226 T3SS^WT^ strain, although the results were not statistically significant (Fig. 4C). Complementation was observed in an arabinose-inducible manner, as shown in Fig. S2. The combined results from isogenic strains differing in T3SS and/or VopX expression status therefore strongly suggest that VopX is required for the maximum T3SS-dependent increased adherence of Caco-2/BBE cells during AM-19226 infection.

### VopX activity does not affect intestinal epithelial barrier integrity

**Fig 5:**
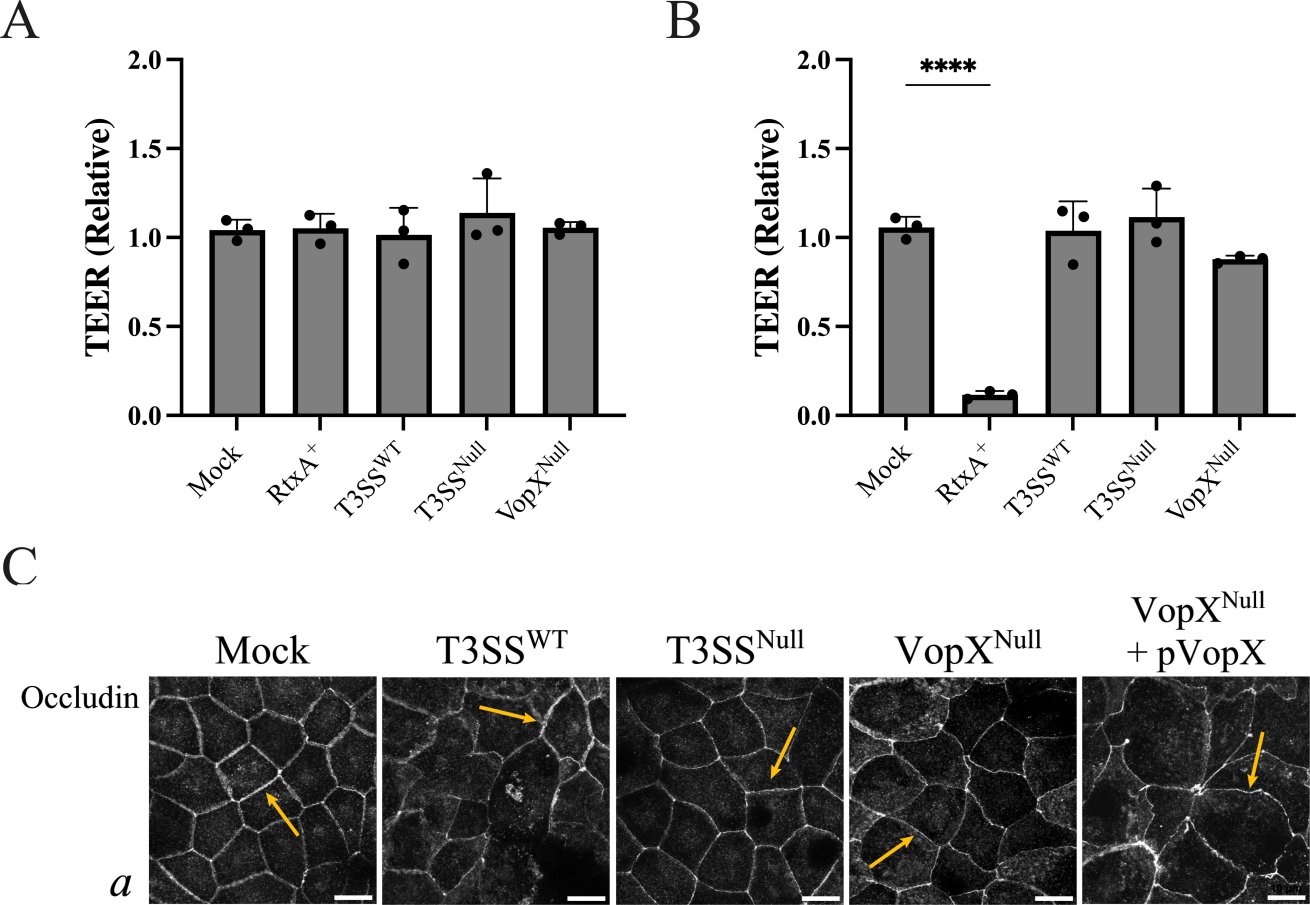
VopX activity does not alter Caco-2/BBE junctional integrity after co-culture at a multiplicity of infection (MOI) of ~10. (A) Relative transepithelial electrical resistance (TEER) assay of Caco-2/BBE cells co-cultured for 3 h at an MOI of 10 with the indicated *V. cholerae* strain or PBS (mock). Data are presented as the average ± standard deviation of three biological replicates. (B) Relative transepithelial electrical resistance assay of Caco-2/BBE cells co-cultured for 3 h at an MOI of ~1,000 with the indicated *V. cholerae* strain or PBS (mock). Data are presented as the average ± standard deviation of three biological replicates. Statistics were generated by ordinary one-way ANOVA with Dunnett’s multiple comparison test. ****P ≤ 0.0001. (C) Three planes, taken at 0.30 μm step size, shown as a maximum intensity projection of differentiated Caco-2/BBE monolayers co-cultured with the indicated AM-19226 strain for 3 h (MOI of ~10 or PBS). Cells were fluorescently labeled for F-actin with phalloidin (red), nuclei with DAPI (blue), and occludin (green). Images show *Z* slices from the basal cell surface. Scale bars represent 10 μm. The study was repeated with similar results.

Differentiated Caco-2/BBE cell monolayers are commonly used to model the barrier function of the intestinal epithelium (56). We hypothesized that VopX-dependent remodeling of the actin cytoskeleton, shown in previous figures, would disrupt epithelial apical junctions and increase permeability of epithelial cell monolayers. Surprisingly, we instead found that co-culture with any AM-19226 strain at an MOI of ~10, regardless of T3SS or VopX status, did not alter epithelial integrity as measured by transepithelial electrical resistance (TEER). Because the AM-19226 strain used in co-culture is deleted for the *rtxA* gene encoding the MARTX toxin (as described in Table 1 and Materials and Methods), previously shown to encode a protein-disrupting junctional integrity, we included MD992, the RtxA+ isogenic parent strain as a positive control for junctional disassembly (57). However, co-culture with the RtxA+ strain did not result in decreased TEER values at an MOI of 10 (Fig. 5A).

When the MOI was increased to ~1,000, Caco-2/BBE cells co-cultured with the RxtA+ strain resulted in a 90% reduction in TEER values, similar to that previously reported for RtxA+ strains (Fig. 5B) (39, 58). We thus conducted co-cultures with the T3SS-isogenic derivative strains at the higher MOI. Co-culture with AM-19226 T3SS^WT^ and AM-19226 T3SS^Null^ strains resulted in TEER values similar to that observed for mock-infected cells. Co-culture with the AM-19226 VopX^Null^ strain resulted in a ~13% reduction in TEER values compared to mock infection, which was not statistically significant. Occludin staining at the apical pole, as shown in Fig. 5C (arrows), appeared similar to that for mock-infected Caco-2/BBE cells and cells co-cultured with the AM-19226 derivative strains, indicative of intact tight junctions. We therefore concluded that under the conditions tested, neither T3SS nor VopX activity dramatically alters tight junction integrity.

### VopX acts as a RhoA guanine nucleotide exchange factor mimic

Importantly, when VopX was expressed in *S. cerevisiae*, we observed cellular phenotypes that also supported a role in modulating actin structures and suggested a protein within the CWI pathway as a possible target of VopX activity. Based on results from our previous genetic studies in yeast (44), we performed a yeast growth inhibition assay using a *S. cerevisiae* strain carrying a vector expressing Rho1 in a GTP-locked conformation from the dominant mutant allele RHO1 Q68H. We observed a 4-log reduction in yeast growth when VopX was co-expressed with RHO1 Q68H (Fig. S3). We thus had several lines of evidence pointing to a role for VopX in stimulating the activity of Rho1, a key yeast protein upstream of CWI MAPK signaling that governs actin-based phenotypes.

**Fig 6:**
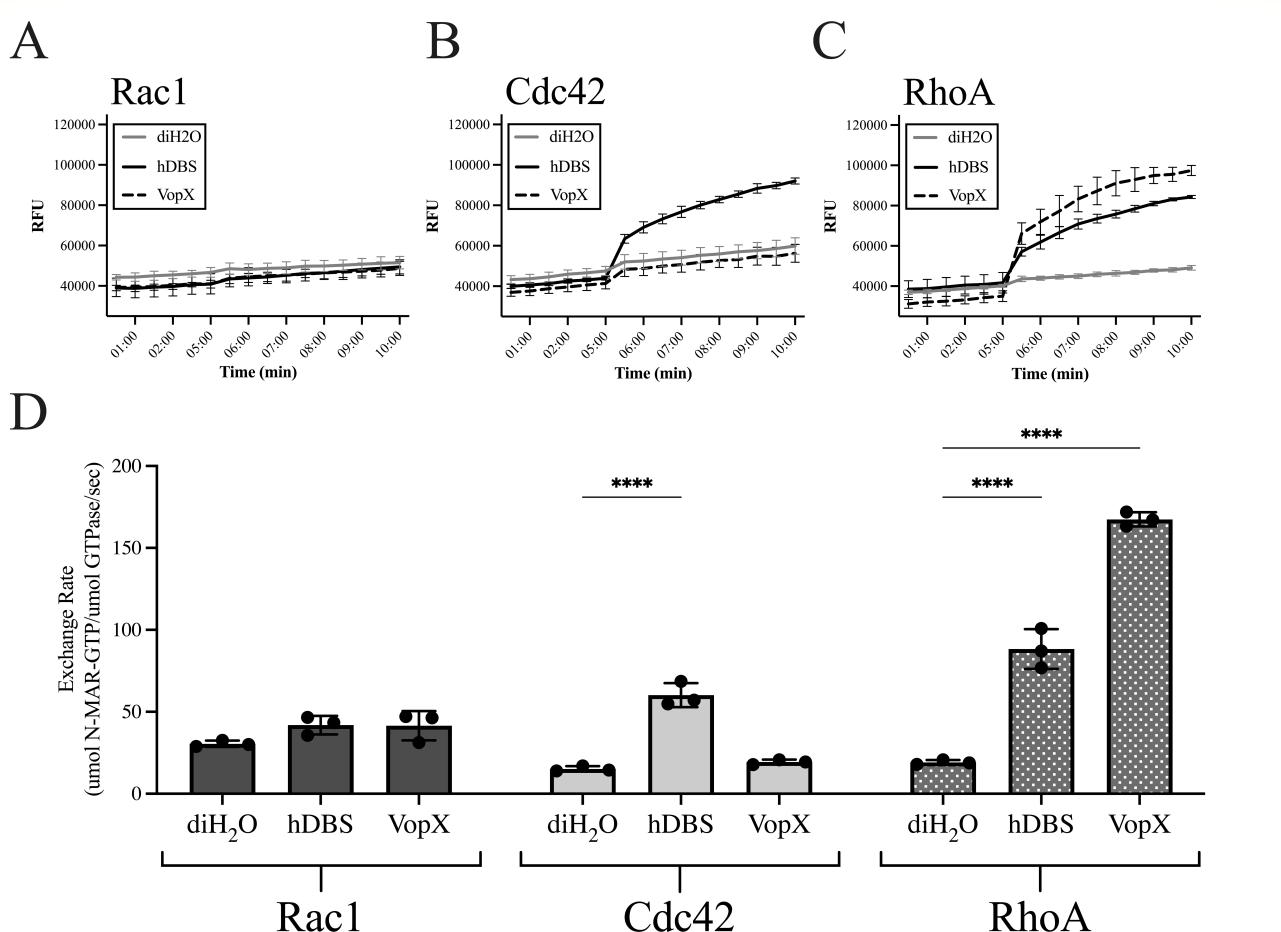
Purified VopX acts as a GEF for RhoA *in vitro*. Relative fluorescence of N-MAR-GTP when combined with purified Rac1 (**A**), Cdc42 (**B**), or RhoA (**C**). The indicated GEF or water was added at *T* = 5:00. Gray lines indicate activity due to diH_2_O; black lines indicate hDBS activity; and dotted lines indicate VopX activity. (D) RhoGEF GTPase exchange rates represented as micromole N-MAR-GTP per micromole GTPase per second. Statistics were generated using two-way ANOVA with Dunnett’s multiple comparison test. ****P < 0.0001. The assay was conducted three times and produced similar results.

RhoA is the human homolog of Rho1. We therefore tested whether VopX targets RhoA activity using purified VopX protein in an *in vitro* guanine nucleotide exchange factor (GEF) exchange assay. The fluorescent nucleotide analog N-methylanthraniloyl-GTP (N-MAR-GTP) was used to measure the activities of three major members of the Rho-family small GTPases as shown in Fig. 6: Rac1 (Fig. 6A), Cdc42 (Fig. 6B), and RhoA (Fig. 6C). As a positive control, we used the purified DH/PH domain of human Dbs (hDBS, “Dbl’s big sister”), which can activate RhoA and Cdc42 but not Rac1 (59–61). Under our experimental conditions, hDBS also activated Cdc42 and RhoA as reflected by the increase of the relative fluorescent unit values, without altering Rac1 activity (Fig. 6A through C). Notably, VopX strongly activated RhoA but had no effect on Rac1 or Cdc42 (compare gray and dashed lines) as shown in Fig. 6C.

Figure 6D shows the average calculated exchange rate for each GTPase when incubated with water as a negative control, hDBS as a positive control, or VopX. The average, basal exchange rates of Rac1, Cdc42, or RhoA (diH_2_O control) were less than 30 µmol N-MAR-GTP/μmol GTPase/s. hDBS is not reported to function as a GEF for Rac1, and the exchange rates were similar to the water control (42 µmol N-MAR-GTP/μmol GTPase/s). The exchange rates of hDBS + Cdc42 and hDBS + RhoA were 60 and 88 µmol N-MAR-GTP/μmol GTPase/s, respectively, and were as expected based on known hDBS specificities. Whereas the average exchange rates of VopX + Rac1 and VopX + Cdc42 were similar to or below that of the water control, the average exchange rate of RhoA in the presence of VopX was 167 µmol N-MAR-GTP/μmol GTPase/s, approximately a two-fold increase compared to that observed for hDBS. We therefore concluded that VopX can function as a GEF specifically for RhoA and that the phenotypes presented in earlier figures could result from VopX-dependent stimulation of RhoA activity.

### VopX can be modeled as part of a RhoA-GEF complex

Secondary structure predictions using Alphafold2 identified six alpha helices formed by VopX amino acid sequences, with a ~55-amino acid disordered N-terminal domain (Fig. S4). The VopX tertiary structure was predicted to be a V-shaped helix bundle, with large helical regions separated by unstructured turns. The predicted local distance difference test (pLDDT) run by Alphafold2 is used to estimate the local confidence of individual amino acids within a structure. While the residues within the N-terminal domain were predicted with low confidence (50 < pLDTT < 70, orange), residues within the V-shaped helical regions were predicted with high confidence (pLDDT >70, blue) and appear grouped into two anti-parallel, three-helix bundles connected at a ~60° angle. The overall predicted template modeling score (pTM) and pLDDT values for VopX structural prediction were 0.662 and 74, respectively.

**Fig 7:**
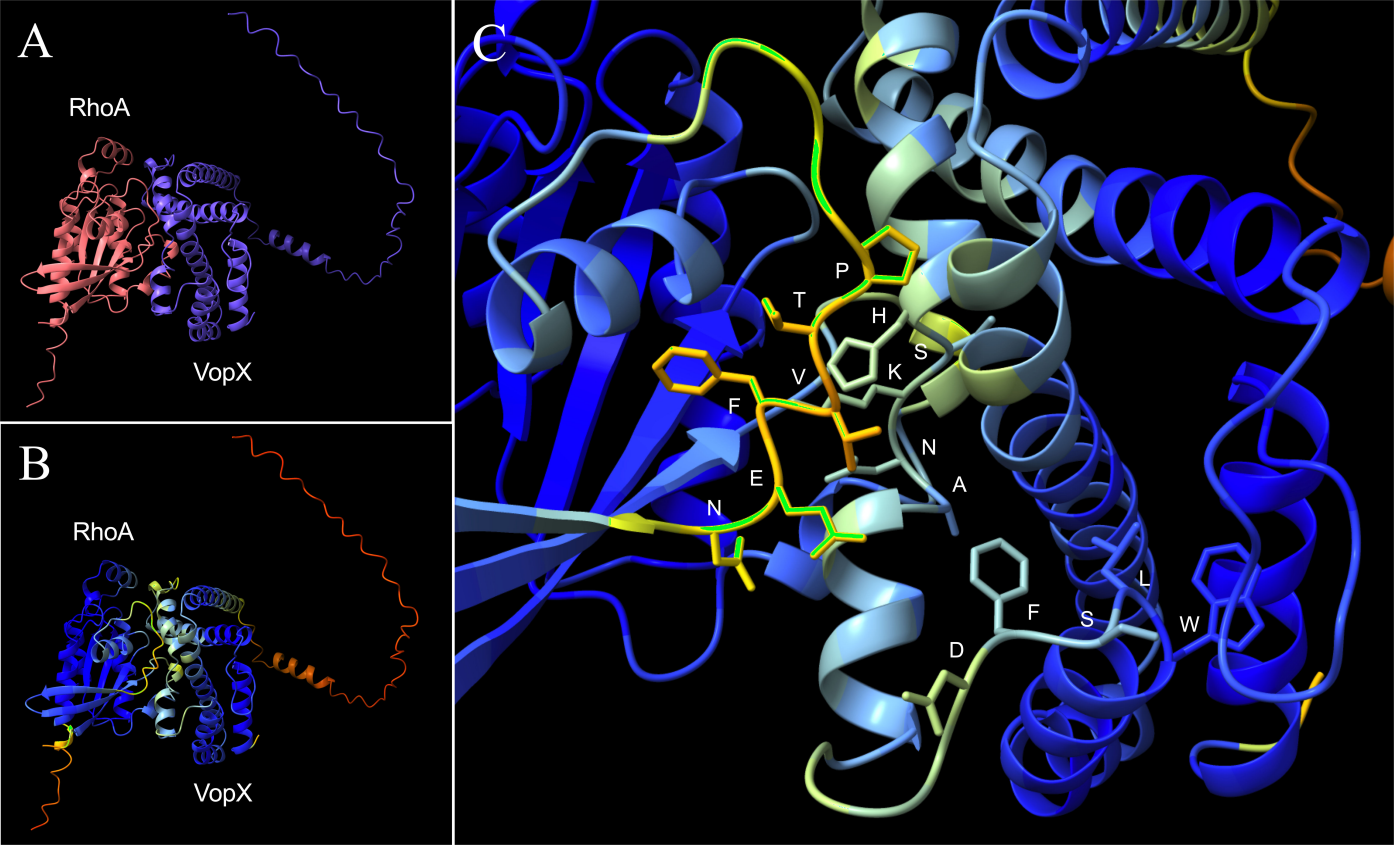
VopX and RhoA can be modeled to form a heterodimeric complex. ColabFold-AF2 modeling of the heterodimeric interaction between VopX and RhoA. (A) RhoA is shown in pink and VopX in purple. (B) Duplicate image of RhoA and VopX colored by confidence level: dark blue, pLDTT >90; blue, 70 < pLDTT < 90; yellow, 50 < pLDTT < 70; orange, pLDTT < 50. (C) Enlarged image showing the amino acids at the site of the protein-protein interface. Atoms from amino acids ^34^PTVFEN^41^ (RhoA), ^148^WLSFD^152^ (VopX), and ^166^ANKSH^170^ (VopX) are shown at the predicted protein-protein interface.

We also used ColabFold with AlphaFold2 to model a heterodimeric protein complex including RhoA and VopX, with results shown in Fig. 7. Panel A depicts RhoA in pink and VopX in purple to clearly differentiate between the two peptides and to illustrate the alpha-helical nature of VopX as described above. Panel B shows the same proteins, with residues colored by the predicted confidence with the highest residue confidence (pLDTT > 90) in dark blue and lowest residue confidence in red (pLDTT < 50). The pTM and interface predicted template modeling values were 0.77 and 0.82, respectively. Overall, the majority of residues in the RhoA-VopX complex were modeled with high confidence, and the model revealed a putative protein-protein interface, enlarged in Fig. 7C. The atoms shown at the proposed interface are ^166^ANKSH^170^ (VopX) and ^35^VPTVFEN^41^ (RhoA). The VopX residues at the proposed interface reside within an unstructured loop connecting the two anti-parallel three-helix bundles. Figure S5 shows the predicted aligned error plot associated with the prediction in Fig. 7.

## DISCUSSION

The unique sequence nature of T3SS effector proteins presents numerous challenges for identifying molecular functions and eukaryotic protein targets and underscores the importance of investigating how novel activities alter signaling pathways, disrupt homeostasis, and lead to pathogenic outcomes. T3SS-positive *V. cholerae* strains present an additional opportunity to understand how a clinically similar disease results from TCP/CT-independent mechanisms. Several effectors required for colonization and secretory diarrhea have been identified thus far using animal models, but only a few effector activities have been determined at the molecular level.

We initially identified the host actin cytoskeleton as a putative target of VopX activity using *S. cerevisiae* as a simple model system (38, 44). In the present work, we used two *in vitro* model systems to experimentally ask if VopX targets the mammalian actin cytoskeleton. *In vitro* systems have several advantages for investigating molecular functions, including the tight control of experimental conditions and ease of reproducibility. When VopX was expressed in HeLa cells, we observed unique morphological phenotypes, including cell rounding/constriction, suggesting major VopX-mediated cytoskeletal remodeling (Fig. 1). We also observed VopX-dependent reorganization of the actin cytoskeleton in polarized Caco-2/BBE intestinal cell monolayers (Fig. 2). The actin cytoskeletal remodeling was accompanied by a significant reduction in cell height and loss of columnar morphology. In addition, we found that the spatial redistribution of the cytoskeletal structures manifested as loss of apical and lateral actin filaments and an increase in basal actin stress fibers enriched in myosin IIB (Fig. 3). The collective data strongly support the conclusion that VopX promotes intestinal cell stress fiber formation during infection.

Zyxin staining provided further evidence that VopX mediates focal adhesion formation (Fig. 4). One function of stress fibers is to increase cell adherence to extracellular matrix at the basal surface. Mammalian cells in serum-rich media predominantly use fibronectin as an extracellular matrix component in adherence, consistent with the presence of fibronectin as a major component of intestinal tissues. We therefore asked whether VopX activities might alter host cell-matrix adhesion, which was then demonstrated directly using the cell adhesion assay (Fig. 4).

One possible functional significance of the VopX-mediated increase in epithelial cell-matrix adhesion relates to intestinal cell and tissue homeostasis. The intestinal epithelium is a dynamic structure with continuous migration of differentiating cells along the crypt-villous axis and subsequent cell extrusion from the villous tip into the gut lumen. Cell extrusion can serve as a protective mechanism that helps shed intestinal epithelial cells colonized by pathogenic bacteria (62). It is likely that the VopX-dependent increase in matrix adhesion of intestinal epithelial cells is beneficial to the bacteria since it might delay shedding of infected epithelial cells and sustain bacterial colonization of the host.

Our data suggest that mammalian RhoA is the direct target of VopX activity. Our conclusion is based on the results of the GTPase exchange assay (Fig. 6) and the VopX-dependent cell rounding phenotype, which is a common consequence of RhoA activation (Fig. 1). Interestingly, at the same micromolar concentrations, the rate of GTPase exchange observed in the presence of VopX was greater than that observed for the human GEF Dbs. RhoA activation is therefore the most likely molecular explanation for the actin-based phenotypes we consistently observed in all model systems.

It is notable that in the same assay, VopX was unable to activate the Rho family GTPases Cdc42 and Rac1, highlighting the specificity of VopX and RhoA-mediated signaling pathways. Despite converging on host actin cytoskeletal modifications, Rho family GTPases Rho, Rac, and Cdc42 regulate three separate signal transduction pathways resulting in actomyosin contractility and formation of lamellipodia and filopodia (63–66). Phenotypes resulting from RhoA activation include actin filament polymerization and increased actomyosin contractility that result in stress fiber assembly and FA formation (67). FA provides a structural link to the extracellular matrix and regulates cell contractility and migration. Zyxin is a critical component of FA and has binding sites for actin, as well as NMII, α-actinin, and GEFs (54, 67). Thus, the VopX specificity for RhoA suggests that actomyosin contractility is the intended outcome of VopX activity and may explain the actin-rich membrane protrusions observed in VopX-expressing HeLa cells.

Previously published co-culture studies of T3SS-expressing non-O1/non-O139 *V. cholerae* strains with the Caco-2 cell line demonstrated T3SS-dependent reduction in TEER, suggesting a loss of barrier integrity (39). Unexpectedly, in our initial experiments, we did not observe a significant change in the TEER during co-culture with strain AM-19226 at an MOI of 10. Of note, we did observe reduced TEER values after co-culture with the VopX^Null^ strain at an MOI of ~1,000, consistent with published experimental parameters and suggesting VopX is important for maintaining barrier integrity at high cell density. High bacterial cell density may recapitulate the bacterial load during human infection, since infected patients have been shown to excrete approximately 10^8^ *V. cholerae* cells per milliliter in stool samples (68).

How VopX activities are coordinated with other effectors to result in colonization and secretory diarrhea remains to be addressed. T3SS effector activities often work in conjunction with one another or offset unintended consequences of signal pathway perturbations (69). As described earlier, strain AM-19226 translocates two other effectors that have been shown to target the host actin cytoskeleton, VopF and VopM (39, 70–72). Both VopF and VopM deletion strains are attenuated for murine colonization *in vivo*, VopM dramatically so, suggesting that remodeling the host cytoskeleton is important for establishing bacterial colonization (43). Our previously published results indicate that VopX is not required for infant mouse colonization. However, we have not tested the VopX null strain for defects in adherence to Caco-2/BBE cell monolayers, which might further reveal a role for VopX early in infection. One interpretation of our current data frames VopX activity as important for later stages of infection, where promoting host cell attachment to the intestinal tissue maintains and stabilizes the ability of microcolonies to be supported later during infection.

Effectors from other pathogens that act as RhoA GEF mimics provide additional evidence illustrating pleiotropic effects of RhoA perturbation. The pathogenic *E. coli* T3SS effector EspM also activates RhoA and leads to the formation of global parallel stress fibers that result in altered cell morphology (69, 73). The downstream consequence of RhoA activation is to inhibit bacterial pedestal formation, suggesting that antagonistic activities of EspM and EspF (which enhance actin pedestal formation) exert balancing functions to avoid severe disruption of intestinal barrier functions. Interestingly, the T33S2 of *Vibrio parahaemolyticus* encodes an effector, VopO, that functions to activate GEF-H1, a RhoA GEF (74).

Characterization of numerous effectors from multiple species has led to recognition of the “SopE/WxxxE family” of T3SS effectors, which function by mimicking the activity of eukaryotic GEF proteins. After the first description of the WxxxE family of bacterial effectors by Alto et al. (26), the conserved motifs were identified in many other T3SS-expressing pathogens (26, 28, 75, 76). Structural studies investigating WxxxE protein family interactions with human GTPases revealed conformational changes around the GTPase nucleotide binding site nearly identical to the Dbl family of eukaryotic GEFs, highlighting the ability of effectors to act as molecular mimics and subvert eukaryotic pathways for bacterial benefit (75, 77). Despite the lack of amino acid similarity to known proteins, VopX may harbor novel or variant GEF sequence motifs which would place it within the WxxxE family of bacterial effectors (78).

VopX is predicted to fold into a V-shaped structure consisting of two three-helix bundles, which is common for other WxxxE- and SopE-like effectors (Fig. S4) (76, 78). The amino acid sequence of VopX ^148^WLSFD^152^ resembles the WxxxE motif with an aspartic acid residue substituted for the glutamic acid residue. The amino acids in the VopX putative WxxxE motif region most closely resemble *Shigella* IpgB1, suggesting that the consensus sequence may increasingly diversify as additional GEF mimics are identified. VopX also contains the peptide sequence ^194^ERRKAQ^199^, which bears resemblance to the D/ExxxAQ motif found in the catalytic loops of EspM, IpgB2, and other family effectors (76). Notably, ColabFold-AF2 heterodimeric structural prediction of VopX and RhoA revealed a putative protein-protein interface between VopX ^166^ANKSH^170^ and the switch I region of RhoA ^34^PTVFEN^41^, consistent with the reported protein-protein interface for hDBS and RhoA, and WxxxE effectors and RhoA (76, 79).

Our collective results identified VopX as a molecular mimic for RhoA GEFs. Along with other molecular mimics, the exciting discovery of VopX function highlights that structural and functional conservation between bacterial and eukaryotic proteins is an important pathogenic strategy for manipulating host pathways, ultimately disrupting homeostasis (73, 80, 81). Notably, VopX sequences are unique to *Vibrio* strains and restricted to limited T3SS2 clades. One potentially interesting interpretation is that T3SS effector proteins, their target specificity, and the associated molecular consequences have thus evolved toward requirements for optimal, species-specific pathogenesis.

Compared to TCP/CT-positive strains, T3SS-positive strains and the associated effector protein repertoires therefore represent a fundamentally different pathogenic strategy. Thus, *V. cholerae* is an organism that employs multiple and dramatically different virulence mechanisms, which interestingly and perhaps surprisingly cause clinically similar outcomes (82). Although we do not fully understand the complete set of T3SS activities or mechanisms leading to host intestinal colonization, we identified VopX as a third protein translocated by the *V. cholerae* T3SS having activities that influence host cell actin dynamics. Whereas Vops F and M directly interact with actin, VopX instead alters upstream signaling pathways that may serve to stabilize epithelial cells and sustain the colonization niche. Determining how the GEF activity of VopX contributes to *V. cholerae* pathogenesis therefore expands not only our understanding of the importance of the host cytoskeleton in T3SS-mediated pathogenesis but also our understanding of how human cells respond to pathogens to maintain homeostasis.

Finally, our collective work exemplifies the power of using yeast-based genetic approaches for initial studies aimed at identifying putative effector targets and phenotypes. Protein and pathway conservation between higher and lower eukaryotes informed targeted approaches for cell biology and molecular investigations using mammalian systems, where the combined results ultimately led us to identify a novel effector protein function. Given that there are unique effector proteins in *Vibrio* and many other species, such approaches seem warranted for efforts aimed at discovering new molecular pathogenic mechanisms.

## MATERIALS AND METHODS

### Bacterial strains, growth conditions, plasmids, and *in silico* analyses

Bacterial and yeast strains used in this study are listed in Table 1. *Escherichia coli* and *Vibrio cholerae* strains were maintained as frozen stocks at −80°C in Luria-Bertani (LB) broth containing 25% glycerol. *E. coli* strains DH5α or DH5αλpir were used for transformation and plasmid propagation. For *E. coli* and *V. cholerae*, ampicillin and streptomycin were each used at 100 µg/mL. Yeast strains were grown in synthetic complete media supplemented with dextrose (SCD) to a final concentration of 2% or synthetic complete media supplemented with galactose (SCG) to a final concentration of 2%. The parent strain for the VopX^Null^, T3SS^WT^, and T3SS^Null^ strains (AM-19226Δ3) was deleted for the genes encoding non-T3SS virulence proteins with cytotoxic effects (*hap*, *hlyA*, and *rtxA)* as previously described (20, 38). Plasmids were transferred to *V. cholerae* by standard electroporation or conjugation methods. *V. cholerae* cultures were grown in LB, and L-arabinose was added to *V. cholerae* cultures at concentrations indicated as needed (Table 2).

**TABLE 1:** List of strains used in this study^*a*^

Strain	Genotype/description	Reference
*V. cholerae*		
MD992	AM-19226 R^−^ M^+^ Str^r^	(38)
AAC155	MD992 Δ*hap*Δ*hlyA*Δ*rtxA*	(43)
AAC330	MD992 Δ*hap*Δ*hlyA*Δ*rtxA*Δ*vcsN2*	(43)
JC9	AAC155 *vopX*′	(41)
VopX^Null^	AAC155 *vopX*′ pBAD18	This study
VopX^Null^ + pVopX	AAC155 *vopX*′ pBAD18*-VopX*	This study
T3SS^WT^	MD992 Δ*hap*Δ*hlyA*Δ*rtxA* pBAD18	This study
T3SS^Null^	MD992 Δ*hap*Δ*hlyA*Δ*rtxA*Δ*vcsN2* pBAD18	This study
*E. coli*		
DH5α	F−Φ80 d*lac*Δ*M15*(*lacZYA argF* Δ*U169*) *endA1 recA1hsdR17 deoR thi-1 supE44 gyrA96* (Nal^r^) *relA1*	Laboratory stock
DH5αλpir	F−Φ80 d*lac*Δ*M15*(*lacZYA argF* Δ*U169*) *endA1 recA1hsdR17 deoR thi-1 supE44 gyrA96* (Nal^r^) *relA1* λ*pir*	Laboratory stock
*S. cerevisiae*		
BY4742	MATα *his3-Δ1 leu2-*Δ*0 lys2-*Δ*0 ura3*-Δ0	(44)
RHO1 Q68H	BY4742, *RHO1* Q68H allele under the control of the GAL1 promoter	(83)
PKC1 R398A	BY4742, *PKC1* R398A allele under the control of the GAL1 promoter	(84)

^*a*^ Kan^r^, kanamycin resistant; M^+^, methyltransferase positive; R^−^, type II restriction endonuclease deletion; Str^r^, streptomycin resistant.

**TABLE 2:** List of plasmids used in this study^*a*^

Plasmid	Description	Reference
pBAD18 (GW)	Gateway compatible expression vector, arabinose-inducible promoter, Amp^r^	(41)
pBAD18-VopX	VopX expression vector, Amp^r^	This study
pDONR201	Gateway cloning donor vector, Kan^r^	Invitrogen
pBG1805-VopX	*E. coli*-yeast 2µm shuttle vector, P_GAL1_, Amp^r^, URA3	(38)
pBG1805-Yal069W	*E. coli*-yeast 2µm shuttle vector, P_GAL1_, Amp^r^, URA3	(38)
pVOTE-VopX-HA	VopX expression vector, encoding T7 RNA polymerase, Amp^r^	This study
pF13L-HA	Vaccinia virus F13L gene with HA epitope tag cloned into pcDNA3, Amp^r^	(85)
pGFP	eGFP gene cloned into pcDNA3, Amp^r^	Laboratory stock

^*a*^ Amp^r^, ampicillin resistant; Kan^r^, kanamycin resistant.

pBAD18-VopX was constructed using Gateway technology that resulted in VopX coding sequences under control of the arabinose promoter. pVOTE-VopX-HA was constructed using standard restriction enzyme cloning methods, which resulted in VopX sequences with a C-terminal HA tag under control of the T7 promoter. Details will be provided upon request.

SnapGene (SnapGene software) was used for *in silico* sequence analysis and manipulations. ColabFold-AF2 was used to predict the structure of a VopX and RhoA heterodimer with three recycles per model (86, 87). Models were processed using ChimeraX version 1.8 (88).

### Mammalian cell lines and culture conditions

HeLa cells were obtained from American Type Culture Collection (ATCC) and maintained in Dulbecco’s Modified Eagle Medium (DMEM, 4.5 g/L glucose, sodium pyruvate) supplemented with 1% L-glutamine (L-glu, Gibco), 10% fetal bovine serum (FBS, GeminiBio), and 1% pen-strep (PS, Gibco) at 37°C with 5% CO_2_. Caco-2/BBE cells (ATCC) were maintained in DMEM (4.5 g/L glucose, sodium pyruvate) supplemented with 1% L-glutamine and 10% FBS at 37°C with 5% CO_2_, unless otherwise indicated. To generate polarized monolayers, Transwell membrane inserts (Corning, 6.5 mm/0.4 µm Pore) were seeded with 1 × 10^5^ Caco-2/BBE cells per insert in DMEM (1% L-glutamine, 10% FBS) and incubated at 37°C, 5% CO_2_. Apical and basal compartment media were replaced every 48 h for 14 days. Before performing co-culture assays, the integrity of the cell monolayers was monitored by TEER measurement in culture medium using EVOM2 (World Precision Instruments). After 14 days, we typically observed TEER values of ~500 ohms × cm^2^ (89). Differentiated monolayers were confirmed by immunofluorescence labeling using an antibody targeting occludin, a marker for mature tight junctions, as described below (90).

### *Saccharomyces cerevisiae* growth inhibition assay

Experiments were performed as described previously (44). Briefly, *S. cerevisiae* strains were grown in SCD-Ura medium at 30°C for 36 h. Cultures were normalized to an optical density at 600 nm of 1, and 10-fold serial dilutions were spotted onto SCD-Ura or SCG media. Plates were incubated at 30°C for 72 h before photographing. *S. cerevisiae* RHO1Q68H and PKC1R398A strains were a generous gift of David Levin (83, 84).

### VopX expression in HeLa cells

Vaccinia virus vTF7-3 and plasmid pVOTE-1 were gifts of Bernie Moss (NIH). HeLa cells were seeded at a density of 2.5 × 10^6^ cells in DMEM (10% FBS, 1% L-glu, 1% PS) on 12 mm glass collagen-coated coverslips (Neuvitro) and grown overnight at 37°C, 5% CO_2_ until they reached 70-80% confluency. vTF7-3 was added to the cells at a MOI of 0.5. After 1 h incubation at 37°C, transfection was performed with 1 µg of pVOTE-VopX or pGFP and 5 µL of polyethylenimine (1 mg/mL), as previously described (85). Cells and virus were incubated in serum-free DMEM at 37°C and 5% CO_2_. After 18 h incubation, cells were fixed with 4% paraformaldehyde for 15 min. Florescence labeling and microscopy are detailed below.

### Transwell Caco-2/BBE co-culture with *V. cholerae*

Caco-2/BBE cells were maintained in Transwell membrane inserts as described above. Twenty-four hours prior to bacterial co-culture experiments, the media were changed to low-glucose DMEM (1 g/L glucose, sodium pyruvate) supplemented with 5% FBS. On the day of the experiment, the culture medium was changed to low-glucose DMEM (1 g/L glucose, sodium pyruvate) with 5% FBS and 0.5% arabinose. *V. cholerae* strains were added at the MOI indicated to the Transwell apical compartment and co-cultured with the Caco-2/BBE cells for 3 h at 37°C. Cells were washed twice with PBS, fixed in 4% paraformaldehyde (PFA) for 15 min at room temperature (RT), and stored overnight in PBS at 4°C. Florescence labeling and confocal microscopy are detailed below.

### Fluorescence labeling

HeLa cells fixed on coverslips were washed three times with PBS before permeabilizing for 15 min with 0.1% Triton X-100 and blocking for 1 h with 10% FBS in PBS (blocking buffer) at room temperature. Coverslips were incubated with rabbit anti-HA (1:500, Roche 11867423001) for 1 h at room temperature followed by three washes with blocking buffer. Coverslips were then transferred to 4′,6-diamidino-2-phenylindole (DAPI) (1:100, lab supply), Alexa Flour 647 Phalloidin (1:200, Invitrogen A22287), and anti-rat Cy2 secondary antibody (1:300, Sigma AP202J) for 1 h at room temperature followed by three more washes with blocking buffer before being mounted on glass slides using ProLong Diamond antifade mounting media (Invitrogen).

Caco-2/BBE monolayers on Transwell membranes used for bacterial co-cultures were permeabilized in 0.1% Triton X-100 for 15 min at room temperature. After washing three times with PBS, membranes were incubated in blocking buffer for 1 h at room temperature. Membranes were removed from the Transwell using a 21G × 1 ½ PrecisionGlide needle (BD Biosciences) and sterile forceps before being stored in 300 µL blocking buffer spots on parafilm. Membranes were transferred to 300 µL spots containing rabbit antibodies against antibodies against AM-19226 (1:1,000, lab supply), NM IIB (1:400, BioLegend 909901), occludin (1:400, Proteintech 13409), or zyxin (1:200, Sigma-Aldrich HPA004835) and incubated for 1 h at RT. Membranes were washed three times with blocking buffer before incubating for 1 h at room temperature with Alexa Fluor 647 Phalloidin (1:200, Invitrogen A22287), DAPI (1:100, lab supply), and Anti-Rabbit Alexa Fluor 488 secondary antibody (1:1,000, Invitrogen A-11008). Membranes were washed three times with blocking buffer before being mounted onto glass slides using ProLong Diamond antifade mounting media (Invitrogen).

### Microscopy and image processing

Labeled HeLa cells were imaged using a Leica DMIRB inverted fluorescence microscope equipped with Image-Pro Plus software (Mediacybernetics) and minimally processed using Fiji (88). Processing included cropping, setting channels to the desired colors, and adjusting brightness. Quantification of cell perimeter was measured from the phalloidin signal of all cells displaying clear cell boundaries.

Labeled Caco-2/BBE cell monolayers were imaged using the Stellaris 5 Laser Scanning Confocal Microscope (Leica). Images were acquired using a ×63 objective oil immersion lens with a working distance of 140 μm, 0.3 μm step sizes, *xy* resolution of 139 nm, and *z* resolution of 235.82 nm. Confocal scope speed was set to 400 Hz, and images were formatted 1,024 × 1,024 with a zoom factor of 3.1. Exact image sizes were 59.52 μm × 59.52 μm with a pixel size of 58.19 nm × 58.19 nm. Repeated experiments used the same acquisition controls and laser intensity. Images were visualized using ImarisViewer version 9.9.1 (Oxford Instruments) and minimally processed using Fiji (91). Processing included Z stack cropping to include only slices with visible signal, setting channels to the desired colors, and creating a maximum *z* projection including the number of slices indicated in the figure legends. Figures were organized using the Fiji plugin QuickFigures (92).

### Transepithelial electrical resistance assay

Caco-2/BBE cells were maintained in Transwell membrane inserts as described above. AM-19226 strains and mutant derivatives were added at the MOI indicated to the apical side of the membranes and co-cultured with the Caco-2/BBE cells for 3 h at 37°C and 5% CO_2_. Transepithelial electrical resistance of Caco-2/BBE monolayers was measured in triplicate at *T* = 0 h and *T* = 3 h using EVOM2 (World Precision Instruments). Resistance of empty filters was subtracted from all measurements.

### Caco-2/BBE adhesion assay

The adhesion assay was modified from previously published studies (93, 94). Caco-2/BBE cells were seeded at a density of 7 × 10^4^ cells/well in a 96-well tissue culture-treated plate (Corning Costar). Bacterial strains were added at an MOI of ~100 and co-cultured for 3 h at 37°C. The plates were sealed in parafilm, inverted, and centrifuged at 4,000 × *g* for 5 m. Remaining cells were washed with 10 µL PBS, fixed in 100 µL 1% PFA for 15 min, washed again with 100 µL PBS, and stained with 50 µL 0.5% crystal violet for 15 min at room temperature. Cells were solubilized in 200 µL 1% SDS and incubated for 15 min at room temperature. The OD_450_ was recorded using a standard endpoint assay using a Synergy H1 Microplate Reader (Agilent).

### RhoGEF GTPase exchange assay

*In vitro* GTPase activity was assessed using a RhoGEF Exchange Assay Biochem Kit (Cytoskeleton, Inc.) according to the manufacturer’s instructions. Relative fluorescence intensity at excitation (485 nm) and emission (535 nm) wavelengths was recorded every 30 s using a kinetic assay protocol in SoftMax Pro (Molecular Devices) with the Synergy H1 Microplate Reader (Agilent). After recording the relative florescence of 1.5 µM GTPase and 0.75 µM N-MAR-GTP for 2 min, purified VopX or hDBS was added to a final concentration of 0.5 µM. Purified VopX was kindly provided by the Lacy Lab at Vanderbilt University and included amino acids 26–252 (amino acids 1–25 were predicted to be structurally disordered and not essential for VopX activity in *S. cerevisiae* [44]). Protein was stored in a solution containing 25 mM HEPES, pH 7, and 300 mM NaCl. Experiments were performed in triplicate. GTPase exchange rates were calculated according to the formula provided by the manufacturer and expressed as micromole N-MAR-GTP per micromole GTPase per second.

## SUPPLEMENTAL MATERIAL

Supplemental Material mbio.03018-24-s0001.docx Figures S1 to S5.

ASM does not own the copyrights to Supplemental Material that may be linked to, or accessed through, an article. The authors have granted ASM a non-exclusive, world-wide license to publish the Supplemental Material files. Please contact the corresponding author directly for reuse.
