# Supplementary material for: VopX, a novel Vibrio cholerae T3SS effector, modulates host actin dynamics
Source: mBio. 2025 Jan 29;16(3):e03018-24. doi: 10.1128/mbio.03018-24 (PMC11898728; doi:10.1128/mbio.03018-24)
Supplement: Supplemental Material — Figures S1 to S5. [file mbio.03018-24-s0001.docx]

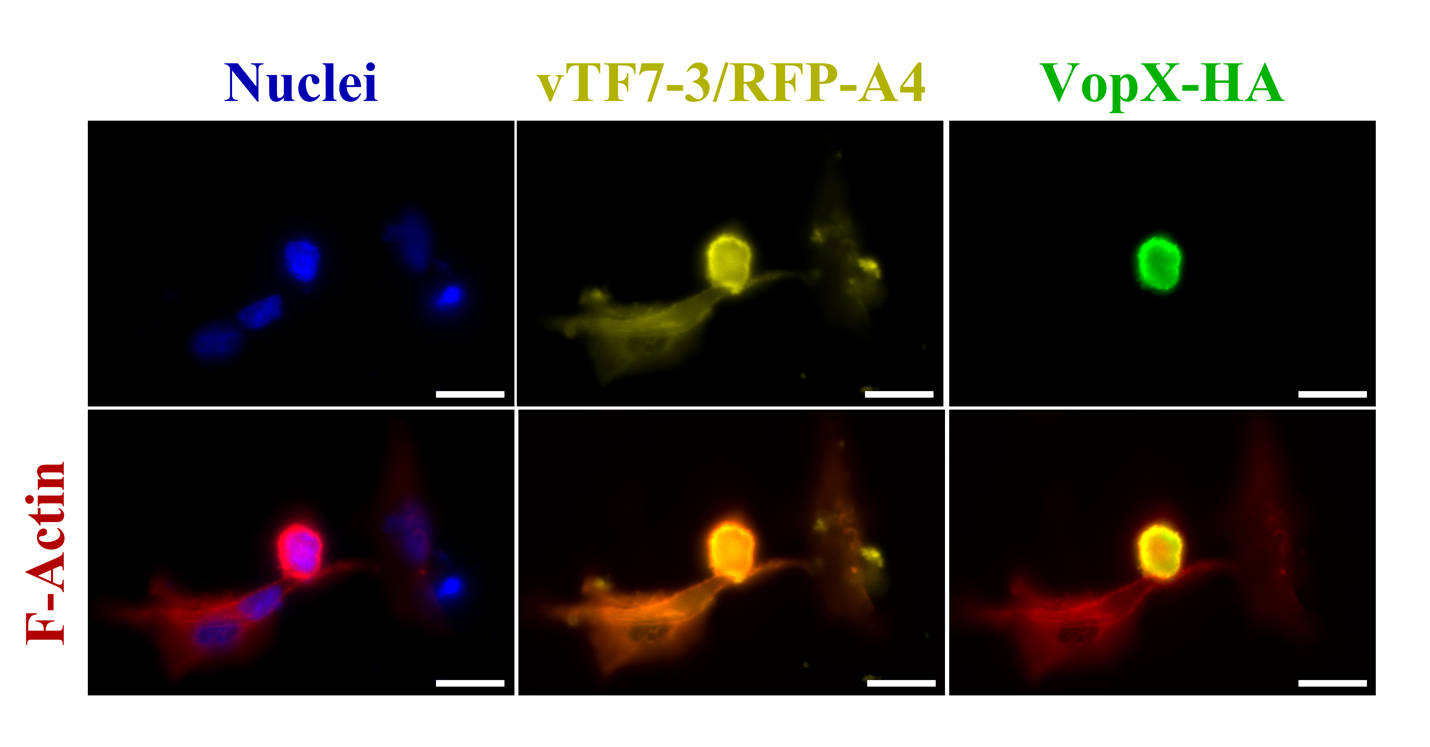


Supplemental Figure 1: VopX overexpression in HeLa cells

Immunofluorescence imaging of HeLa cells infected with Vaccinia virus vTF7-3/RFP-A4 at an MOI of 0.5 and transiently transfected with a vector expressing VopX-HA. Cells were fluorescently labeled for F-actin with Alexa Fluor 647. Individual florescent labels alone are shown in the top row, images merged with phalloidin staining are shown in the bottom row. Scale bars represent 20 μm. The study was conducted three times and produced similar results.


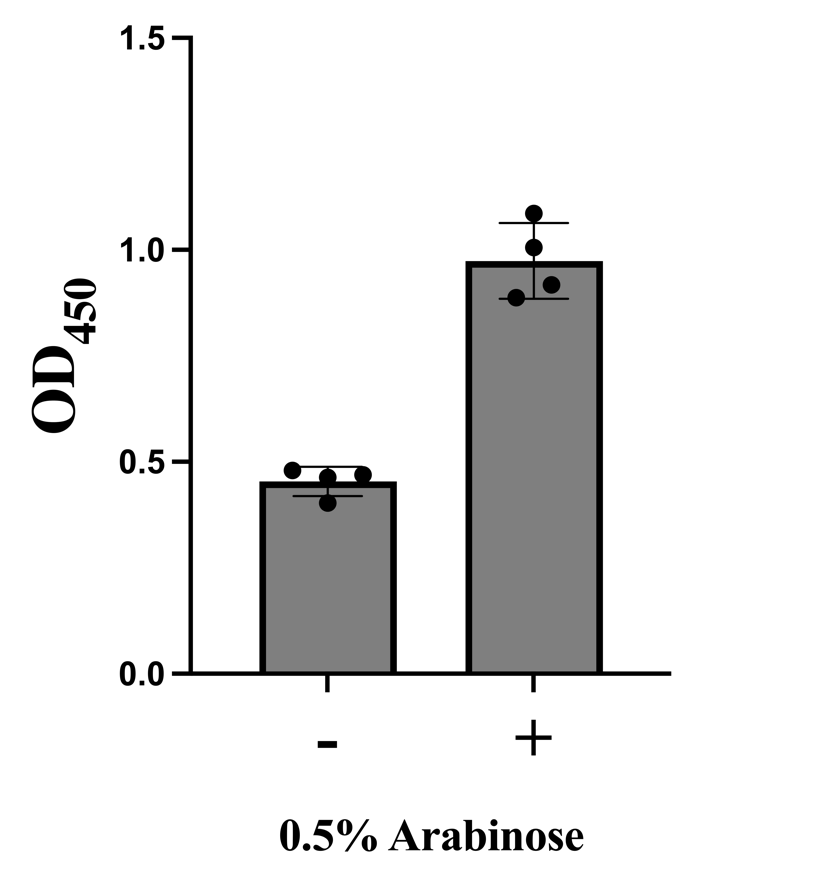


Supplemental Figure 2: Complementation of VopX dependent Caco-2/BBE adherence phenotype following co-culture with strain AM-19226

Following 3 h co-culture of Caco-2/BBE cells with the AM-19226 VopX^Null^ + pVopX strain in the presence (+) or absence (-) of 0.5% arabinose., culture plates were inverted, spun at 4,000 x g and well stained with crystal violet. The optical density at 450 nm of the remaining, stained Caco-2/BBE is shown on the y-axis. Data represent the average of four biological replicates. The study was conducted three times and produced similar results.


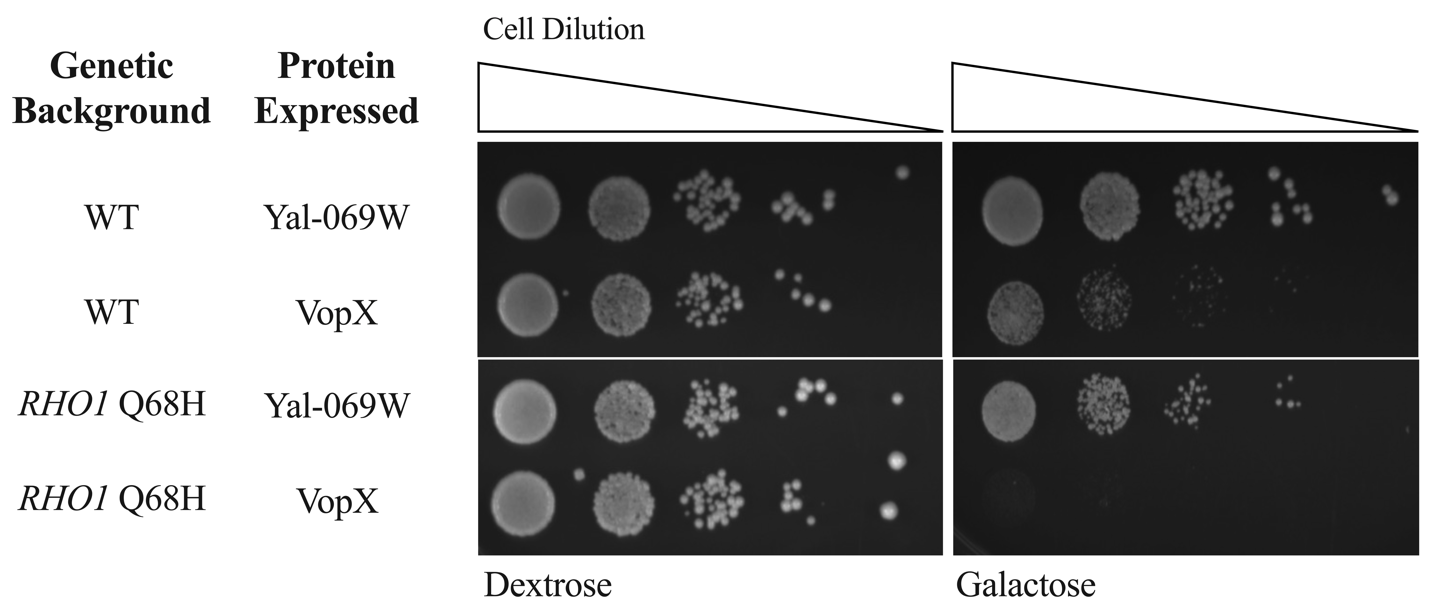


Supplemental Figure 3: Yeast inhibition assay

Yeast growth inhibition assay showing exacerbated VopX-dependent growth defects in the presence of a constitutively active *RHO1* Q68H mutant allele. Strains carry plasmids expressing VopX or Yal-069W, and a plasmid carrying the RHO allele or the empty vector. After growth at 30 ^o^C to saturating conditions, strains were serially diluted 10-fold and spotted on plates containing galactose (expression inducing conditions) and dextrose (non-inducing conditions). The study was conducted three times and produced similar results.

.
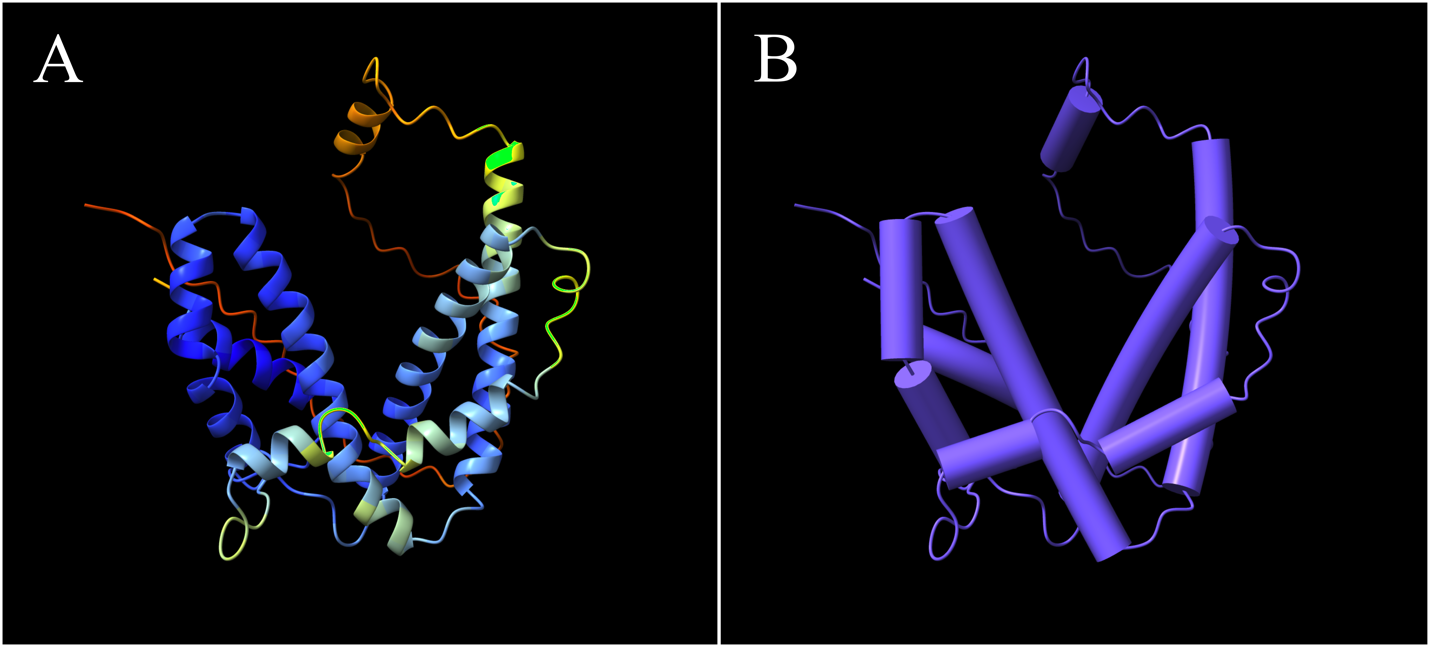


Supplemental Figure 4: VopX Alphafold2 structural prediction

Alphafold2 modeling of VopX. **A –** VopX residues are colored by the prediction confidence level: Dark blue – pLDTT > 90, Blue – 70 < pLDTT < 90, Yellow – 50 < pLDTT < 70, Orange – pLDTT < 50. **B –** Duplicate image of VopX prediction with helical domains represented as cylinders.


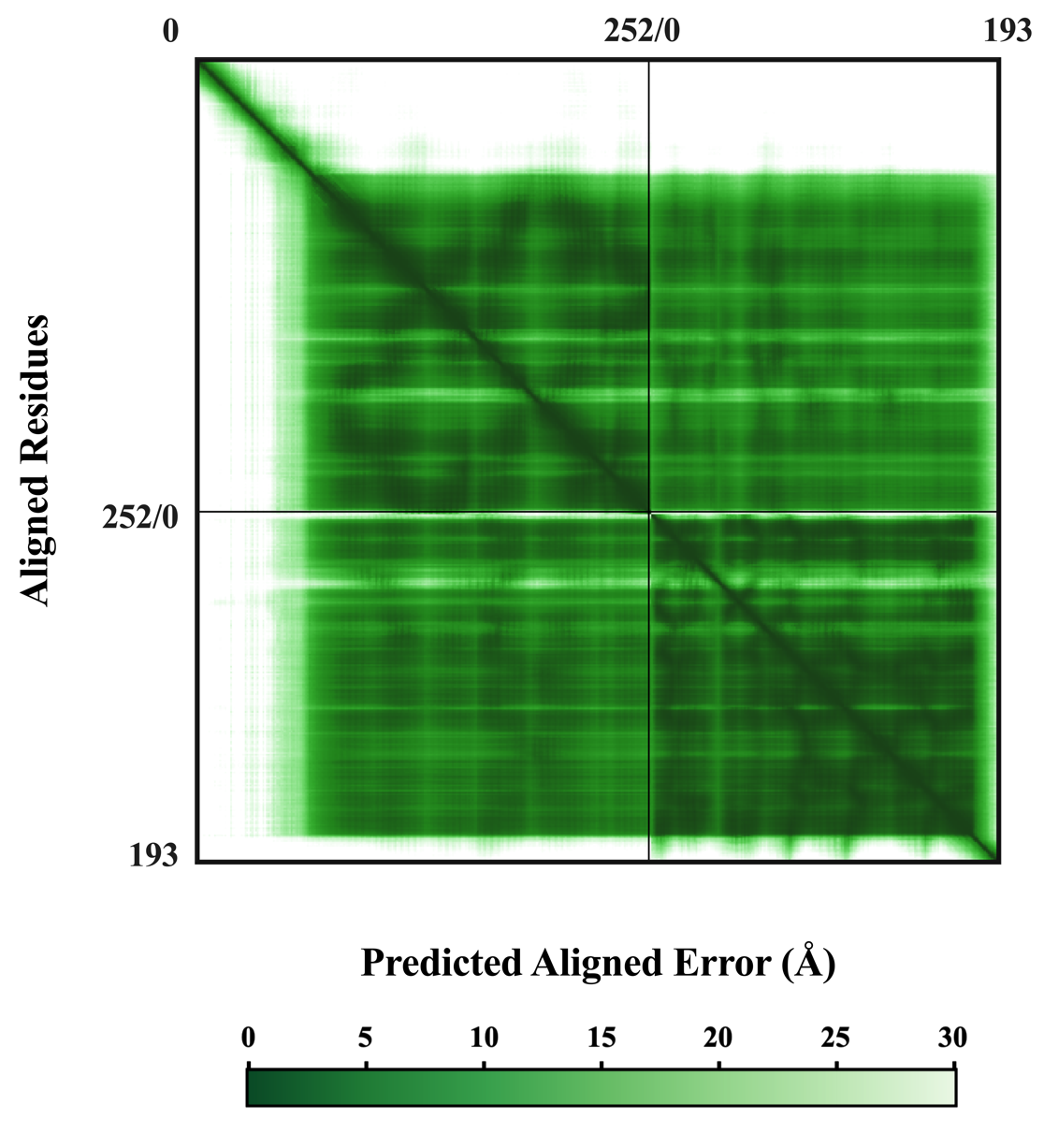


Supplemental Figure 5: Predicted aligned error for VopX and RhoA complex

Domain position confidence scores calculated for ColabFold-AF2 VopX and RhoA complex prediction. Values range from 0 - 30 Angstroms. VopX and RhoA peptide sequences are plotted along the vertical and horizontal axes. Color intensity at each pixel indicates Predicted Aligned Error value (angstroms) for the corresponding pairs of residues.
